# Micromechanical Properties of a New Polymeric Microcapsule for Self-Healing Cementitious Materials

**DOI:** 10.3390/ma9121025

**Published:** 2016-12-20

**Authors:** Leyang Lv, Erik Schlangen, Zhengxian Yang, Feng Xing

**Affiliations:** 1 Micromechanics Laboratory (MICROLAB), Faculty of Civil Engineering and Geosciences, Delft University of Technology, Stevinweg 1, 2628 CN Delft, The Netherlands; L.Lu-2@tudelft.nl (L.L.); Erik.Schlangen@tudelft.nl (E.S.); 2 Guangdong Province Key Laboratory of Durability for Marine Civil Engineering, School of Civil Engineering, Shenzhen University, Shenzhen 518060, China; 3 Department of Civil and Environmental Engineering, Washington State University, P.O. Box 642910, Pullman, WA 99164-2910, USA; zhengxian.yang@wsu.edu

**Keywords:** phenol–formaldehyde, microcapsule, self-healing, nanoindentation, micromehcanical properties

## Abstract

Self-healing cementitious materials containing a microencapsulated healing agent are appealing due to their great application potential in improving the serviceability and durability of concrete structures. In this study, poly(phenol–formaldehyde) (PF) microcapsules that aim to provide a self-healing function for cementitious materials were prepared by an in situ polymerization reaction. Size gradation of the synthesized microcapsules was achieved through a series of sieving processes. The shell thickness and the diameter of single microcapsules was accurately measured under environmental scanning electron microscopy (ESEM). The relationship between the physical properties of the synthesized microcapsules and their micromechanical properties were investigated using nanoindentation. The results of the mechanical tests show that, with the increase of the mean size of microcapsules and the decrease of shell thickness, the mechanical force required to trigger the self-healing function of microcapsules increased correspondingly from 68.5 ± 41.6 mN to 198.5 ± 31.6 mN, featuring a multi-sensitive trigger function. Finally, the rupture behavior and crack surface of cement paste with embedded microcapsules were observed and analyzed using X-ray computed tomography (XCT). The synthesized PF microcapsules may find potential application in self-healing cementitious materials.

## 1. Introduction

Crack-induced deterioration is one of the severest threats to the safety and durability of cementitious structures, which gives rise to large amounts of rehabilitation work, associated costs and waste of resources every year. A recent estimate shows that, in the Netherlands, one third of the annual construction budget was allocated to the inspection, maintenance and repair of those existing buildings, excluding the cost for reconstruction. In recent years, inspired by the phenomenon of tissue regeneration in biology, microcapsule-based self-healing cementitious composite has gained a great popularity in the civil engineering field [1,2,3,4,5,6,7]. These mechanically triggered capsules are placed in the cementitious structure during the process of concrete mixing. Once a crack occurs in the structure (a mechanical trigger), pre-embedded capsules that are located along the path of the crack are ruptured, and the self-healing function of cementitious composites is achieved through the release of repairing chemicals in the region of damage. Most of the healing agents are susceptible to the surrounding environment. Thus, a better protection to the healing agent means a higher healing efficiency. Over the past decade, research has focused on developing a practical container for the healing agent. This resulted in a series of mechanical trigger microcapsules, which yield promising application potential. Thanks to its excellent water and chemical resistance as well as a low flammability and toxicity, phenol–formaldehyde (PF) resin has been suggested as a promising component of the polymer–cement composites to be used in the civil engineering field [8,9]. Compared to other commonly used organic shell materials such as urea–formaldehyde (UF) resin [10,11] and melamine–formaldehyde (MF) resin [12,13], PF resin possesses higher stiffness, brittleness and tunable physical and mechanical properties. Therefore, the healing agent with a PF resin shell has higher stability in cementitious materials and the possibility to be triggered by crack propagation.

It is known that the realization of the self-healing function largely depends on the trigger mechanism of the microcapsules, which will further influence the self-healing efficiency of the system [14,15]. A good understanding of the mechanical properties of microcapsules is crucial in order to fully understand the trigger behavior and their performance in their target service environment. Meanwhile, the obtained intrinsic mechanical properties of microcapsules such as elastic modulus and rupture stress can be applied to numerical simulations and form a basis for designing applications in the future. In previous studies, mechanical characterization of microcapsules is normally performed by using a cone tip or two parallel plates [16,17,18,19]. While, in most cases, microcapsules inside the cement paste are not ruptured by compression stress, but by the tensile stress which initiates the propagating crack. Thus, the data acquired by using a flat tip indenter cannot be used to evaluate the mechanical properties and rupture behavior of microcapsules that fail under a mechanical trigger. A method to mimic and analyse the performance of microcapsules that are hit by a sharp crack tip is still necessary.

Moreover, an inevitable problem that hinders the way to achieve a sound self-healing effect in reality, is the low trigger ratio of microcapsules. It is ideal that microcapsules in the self-healing cementitious composite can take the proper action to different scale deterioration accordingly. This means that the microcapsule should have the ability to sense and further react to different types and strengths of mechanical triggers. To achieve the multi-sensitive function, a feasible way is to prepare microcapsules with different trigger strength, so that the microcapsules can deal with different local strengths of material.

In the present paper, poly(phenol–formaldehyde) (PF) microcapsules containing a healing agent with excellent dispersibility were prepared by in situ polymerization for self-healing application in cementitious materials. The surface morphology of microcapsules was observed by an optical microscope (OM) and an Environmental scanning electron microscope (ESEM). The diameter of microcapsules was successfully controlled and further graded into a multiple distribution range without overlapping. The relationship between shell thickness and diameter was measured. Nanoindentation was applied to measure the micromechanical properties of the microcapsules and the whole process was recorded under video camera. A berkovich tip was used to mimic the behavior of cracking to get the rupture force of PF microcapsules. Despite the scatter, a significant trend can be observed in the dependence of the rupture force of PF microcapsules on the gradation of microcapsules with particle size distribution ranges 50–200, 200–400 and 400–600 μm. In addition to the mechanical testing, X-ray computed tomography (XCT) was used to visualize the distribution and the rupture status of the microcapsule.

## 2. Materials and Methods

### 2.1. Materials

Phenol, formaldehyde (37%), Sodium hydroxide (NaOH), hydrochloric acid (HCl), Poly (acrylic acid sodium salt) (PAA-Na) ($M_{w}$ = 1200) and liquid Dicyclopentadiene (DCPD) were used to produce the PF microcapsules. Heat-softening glue was used to fix microcapsules on the specimen. Ordinary Portland cement (CEMI 42.5N) was used to make the cement-paste specimens. Deionized water was used for all the experiments and all chemicals are analytical grade and were used without further purification.

### 2.2. Synthesis of PF/DCPD Microcapsules

#### 2.2.1. Preparation of PF Precondensate

An amount of 5.0 g phenol and 7.5 g 37% formaldehyde solution was mixed in a 100 mL three-necked flask equipped with a reflux condenser. The mixture was stirred at 350 rpm with a two-paddle impeller. An amount of 5 wt % NaOH aqueous solution was used to adjust the pH of the mixture to around 9. Then, the mixed solution was placed into a water bath to react at 90 ${}^{\circ }$C for 90 min.

#### 2.2.2. Emulsion

An amount of 14.0 g DCPD was first added to 200 g surfactant aqueous solution containing 15 g 5% PAA-Na solution under a stirring rate of 1000 rpm. The resulting suspension was continually agitated at 65 ${}^{\circ }$C for 20 min to disperse the DCPD in the water. Upon the completion of the emulsion, the PF precondensate solution and the prepared emulsion were transferred to a 500 mL three-necked flask, followed by mixing for 10 min under a stirring rate of 350 rpm.

#### 2.2.3. Synthesis of Microcapsules

**Figure 1:**
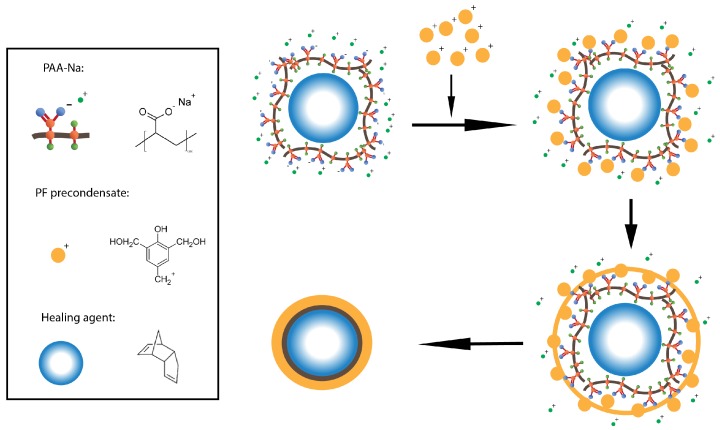
Schematic illustration of the chemical reaction route of PF shell/DCPD core microcapsules via in situ polymerization.

Under the agitation with the double paddle impeller at 350 rpm, 5 wt % HCl aqueous solution was added to adjust the pH of the reaction system to around 1. Then, the system was heated at a rate of 1 ${}^{\circ }$C/min until it reached the polycondensation temperature of 90 ${}^{\circ }$C and was then maintained for 3.5 h. After the reaction has completed, the resulting suspension containing microcapsules was cooled down to room temperature (25 ± 1 ${}^{\circ }$C). The microcapsules were then collected by filtration and then dried in air for 5 h until obtaining a sand-like free-flowing powder. The chemical schematic illustration of this reaction process is shown in Figure 1.

### 2.3. Morphology and Shell Thickness of PF Microcapsules

The morphology and the core/shell structure of the synthesized PF/DCPD microcapsules was characterized by an environmental scanning electron microscope (ESEM) (XL30, Philips, Amsterdam, The Netherlands). All imaging was performed in low vacuum. The rupture pattern and its trigger behavior of the PF microcapsules were observed using an optical stereoscopic microscope (OM) (VHX-600K, Keyence, Osaka, Japan). The shell thickness and diameter of microcapsules were determined from the ESEM micrographs of the cross-section of microcapsules using a commercial dimensional measurement software. Firstly, the diameter of a single microcapsule was record under a microscope. After that, the microcapsule of which the diameter was measured before was then compressed to rupture between two glass sheets. The shell thickness of this microcapsule was determined from the debris of the shell. In this test, a total of 50 microcapsules were randomly selected and the measurement was performed two times on each microcapsule at a different position.

### 2.4. Size Distribution Analysis and Gradation

The mean size and size distribution of synthesized microcapsules produced under different stirring rates was determined using a laser particle size analyzer (LPSA, BT-9300ST, Bettersize Instruments Ltd., Dandong, China). Before the test, microcapsules were washed by deionized water and then placed in a drying box at 60 ${}^{\circ }$C for 24 h. Then, 1 g microcapsules were dispersed by 50 mL deionized water in the analysis box. The values of volume-based mean diameter ($D_{4,3}$) and the value of distribution span (SPAN) were calculated automatically by the instrument. The $D_{4,3}$ and SPAN are defined by the following equation:

(1) $$D_{4,3}=\frac{\sum_{i=1}^{n}d_{i}^{4}}{\sum_{i=1}^{n}d_{i}^{3}}$$

(2) $$SPAN=\frac{D_{90}-D_{10}}{D_{50}}$$

where $D_{90}$, $D_{10}$, $D_{50}$ represent the diameter when the cumulative volume fraction of the measured particles is 90%, 10% and 50%, respectively; $d_{i}$ is the diameter of a single microcapsule and *n* is the number of microcapsules which is measured. The gradation of microcapsules was realized through sieving the microcapsules with different mesh sizes (30, 40, 70). The size distribution of the after-sieving microcapsules was then measured from the OM images of microcapsules using a commercial dimensional measurement software. For each diameter range, at least 50 samples were randomly recorded.

### 2.5. Micromechanical Properties of PF Microcapsules

#### 2.5.1. Elastic Modulus Measurement

Nanoindentation was used to obtain the elastic modulus of microcapsules and shell material. For the measurement of pure shell material, a large block of pure PF resin was made in advance by exactly the same reaction condition as with the preparation of PF microcapsules. Then, the synthesized PF resin block was placed in microtome to cut into a regular flat piece. Before the nanoindentation test, the surface of the sample is ground by four different grinding papers (500#, 800#, 1200#, 4000#). Then, the sample was polished by hand on a lapping table using diamond paste with particle diameter of 6 μm, 3 μm, 1 μm and 0.25 μm. During the measurement, a total of 15 points were selected to test and the indentation depth was set to 2000 nm. The Continuous Stiffness Method was adopted to run the test [20]. The average elastic modulus was determined in the displacement range between 1000 and 1800 nm. Then, the nanoindentation test was performed on microcapsules. Before the test, microcapsules with different diameters were selected and glued on a glass sheet by a heat-softening glue. To be more specific, the glass slide was first placed on a heat plate of 70 ${}^{\circ }$C. Then, the heat-softening glue was smeared on the glass slide and a blade was used to make a flat and uniform glue layer. The microcapsules were then carefully scattered on the slide. After the slide was removed from the heat plate, the glue hardened and the microcapsules were fixed. A diamond Berkovich tip was used for this nanoindentation test. The geometrical characteristics can be found from the referred work of Oliver and Pharr [20]. A quartz standard was indented before and after each test to ensure the accuracy. In this test, the allowable drift rate was 0.15 nm/s, the surface approach velocity was 20 nm/s. The indentation depth was 1000 nm. In this study, the elastic modulus of the shell microcapsule was defined at the linear phase of the load–displacement curve as the mean value in the displacement ranging from 600 nm to 900 nm.

#### 2.5.2. Rupturing-Force Measurements

**Figure 2:**
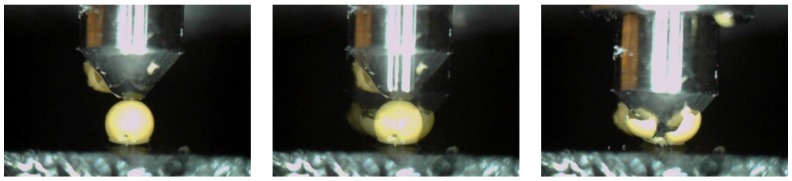
Dynamic process of a microcapsule under the rupturing-force measurement.

In order to investigate the required rupture force of the microcapsule under the strike of a crack, a diamond Berkovich tip was used here as a mechanical trigger. In this test, a total of 50 microcapsules with different size ranges were selected and fixed on the sample holder. Before the test, each microcapsule was photographed from the top by the inset camera of the nanoindentation. The obtained images were used to measure the diameter of each microcapsule. The indent test was performed at the selected physical central of microcapsules . To ensure the rupture of microcapsules, the indentation depth was set to 5000 nm. To visualize the rupture process, a video camera was attached to the sample holder. After the test, the images of ruptured microcapsules were taken again from the top. Figure 2 shows the rupture process of a microcapsule under the hit of a Berkovich indenter.

### 2.6. Investigation in Cement Paste

#### 2.6.1. Preparation of Cement Paste

The cement paste specimens were prepared with a water-to-cement ratio of 0.4. In which, 4% of the cement mass was replaced by PF/DCPD microcapsules . The microcapsules were first dry mixed with cement in a mixer, and then deionized water was added and stirred for one minute to achieve a good workability. After mixing, the fresh mixture was cast in molds to form (a) cubes (10 × 10 × 10 mm^3^) for the compression test; and (b) cylinders (diameter 6.7 mm and length 13.4 mm) for the tensile test and XCT imaging. Then, they were carefully compacted on a vibrating table to reduce the entrapped air. The specimens were demolded after curing under room temperature (RT) and local lab environment for 48 h and cured in a wet chamber at 25 ${}^{\circ }$C and 95% relative humidity. After curing for 28 days, samples were taken out from the curing room. The cement cubes were placed in a compression testing machine to measure the compressive strength. The cement cylinders were fractured by a tensile testing machine to generate a crack in the middle of the specimens. To keep the original position of the fractured sample, before the test, the cylinder sample was wrapped by plastic tape. For each size range of microcapsule (large size, medium size and small size), five samples were tested.

#### 2.6.2. Influence of Incorporation of Microcapsules on Mechanical Properties of Hardened Cement Paste

The influence of microcapsule incorporation on the mechanical properties of hardened cement paste were studied on the cement cubes with embedded microcaspules. Axial Tension-Compression Systems (8872, Instron, High Wycombe, UK) were used to impose a compressive force on the cement cubes with and without microcapsules’ incorporation. The loading speed is set to 0.001 mm/s. A comparison is made between pure cement cubes and microcapsule incorporated cement cubes.

#### 2.6.3. Trigger Behavior of Microcapsules

**Figure 3:**
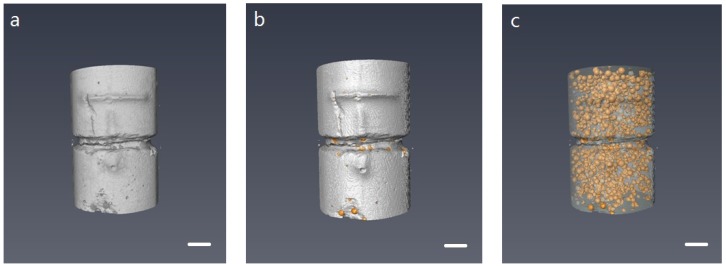
XCT visualization process of microcapsules’ embedded cement paste cylinder including (**a**) volume construction; (**b**) surface generation; and (**c**) transparent adjusting. The scale bar is 2 mm for all three images.

X-ray computed tomography (XCT) scanning technology (Nanotom, GE Inspection Technologies, Lewistown, LP, USA) was applied to investigate the status of microcapsules within hardened cement paste. The raw XCT images were acquired at an acceleration voltage of 50 kV with an exposure time of 4 s and X-ray power of 8 W. The resolution of CT scans is set to 7.5 μm. The final data set of XCT consisted of 720 radiographs of which each image was acquired with a 0.5${}^{\circ }$ rotation. Then, phase retrieval and tomographic reconstruction were performed to improve the boundaries and signals using the software supplied by the manufacturer. A series of reconstructed tomographic images (X–Z plane) were consequently imported into a commercial software (Avizo 9.0) for segmentation and 3D visualization. The first step of the visualization involves converting the 2D image into the software and generating a 3D volume (Figure 3a). After that, a surface generation step is performed on the volume to separate the sample into two materials with different color: Cement matrix (grey), microcapsules (yellow) (Figure 3b). The cement part was then set as transparent so that the status of microcapsules can be clearly seen (Figure 3c).

## 3. Results and Discussion

### 3.1. Morphology of the PF Microcapsules

**Figure 4:**
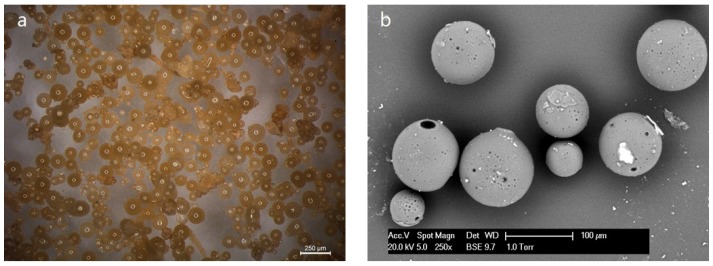
Images of PF microcapsules obtained by (**a**) OM; and (**b**) ESEM.

Figure 4 shows the typical morphology of synthesized PF microcapsules with size distribution between 50 and 150 μm, which was obtained by OM and ESEM. As can be seen from the OM image in Figure 4a, a large amount of microcapsules can be obtained by the proposed method, featuring a clear brown color. The microcapsules possess a regular spherical shape and have smooth surfaces. The ESEM image of microcapsules in Figure 4b shows that the microcapsules are separated from each other without agglomeration. It can be clearly seen from the images that the shell of the microcapsules is homogeneous with pores in it. These pores are believed to have formed during the synthesis process. The vast majority of these pores are embedded pores. The pores will not lead to leaching of the encapsulated material.

### 3.2. Shell Thickness

**Figure 5:**
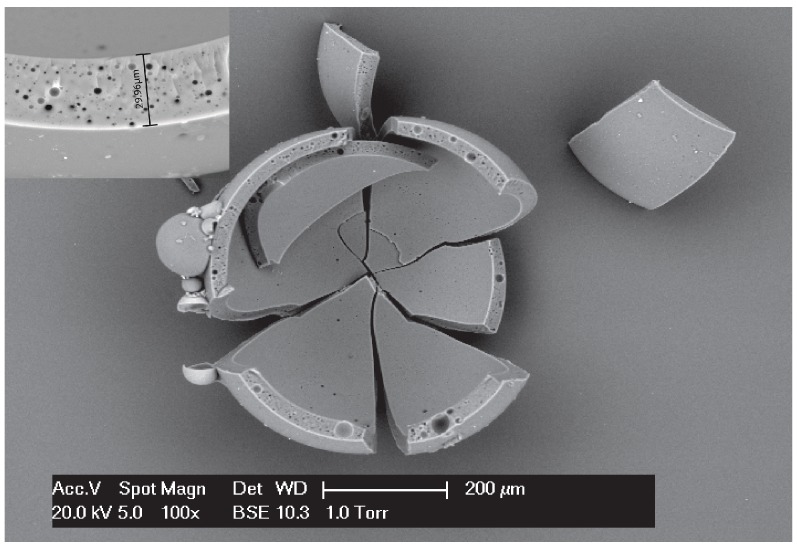
Morphology of a ruptured PF microcapsule. Inset is an enlarged image showing the thickness and structure of the shell of synthesized PF microcapsules.

**Figure 6:**
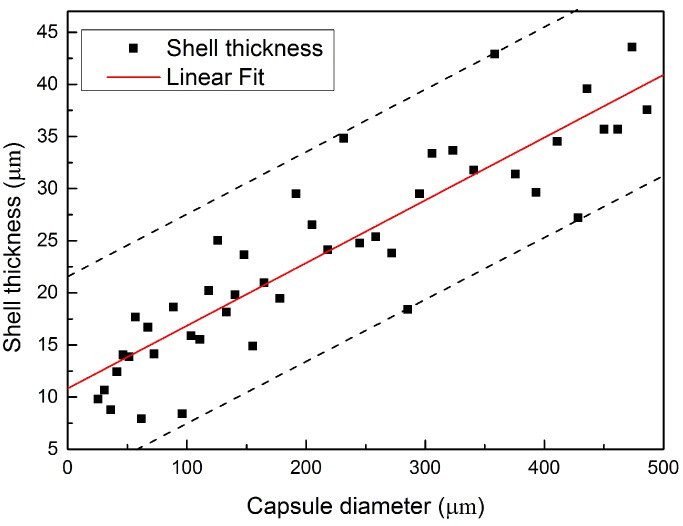
Relationship between the diameter and shell thickness of microcapsules.

The shell thickness and diameter of microcapsules were determined from the cross-section of ESEM images and then calculated by a commercial dimensional measurement software. Due to the brittle nature of PF resin, after the microcapsules were ruptured by compression force, a clear image of a core–shell structure of microcapsule can be observed under microscopy. A typical morphology of ruptured microcapsules is shown in Figure 5. A distinctively low shell thickness/diameter ratio of the microcapsules is obtained. This low ratio offers an ideal storage capacity for the healing agent, which is believed to be a desired feature for self-healing application in cementitious materials. The insert graph is a magnified image of the shell. It shows that the shell thickness of this microcapsules is 29.96 μm. Figure 6 summarizes the relationship between shell thickness and diameter. The result demonstrates that, although the shell thickness to diameter ratio does not show an obvious linear relation and the coefficient of variation is up to 41.24%, the distribution interval of shell thickness is still found to have an increasing trend with the increase of the capsule’s diameter. It should be mentioned that, for better expressing the relationship between the shell thickness and diameter, those microcapsules with shell thickness to diameter ratio at the top or at the bottom 5% of total data were not included in this calculation.

### 3.3. Mean Diameter and Size Distribution Analysis

**Figure 7:**
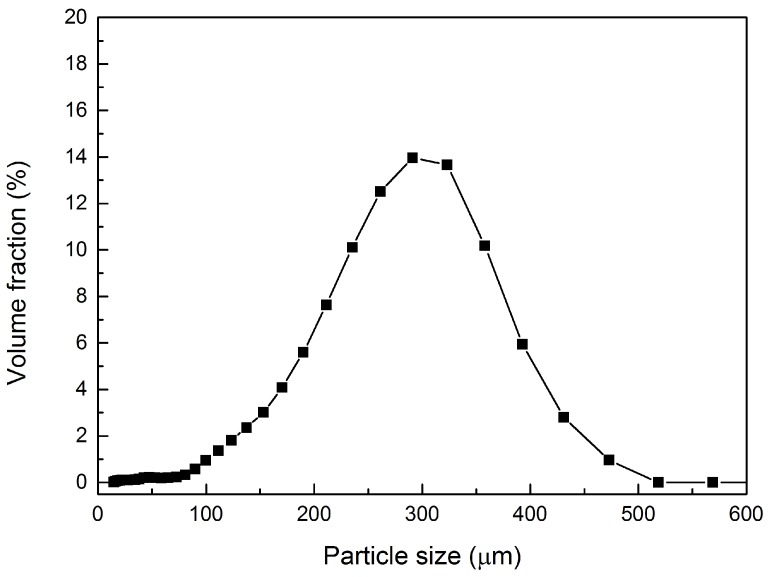
Typical size distribution of microcapsules prepared with a stirring speed at 400 rpm.

**Figure 8:**
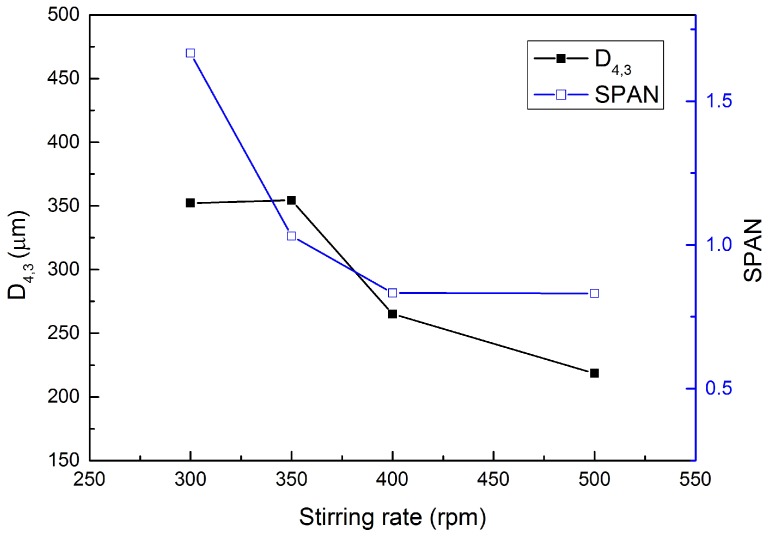
Size distribution of PF microcapsules prepared at various stirring rates (300, 350, 400 and 500 rpm).

The size distribution of synthesized microcapsules was measured and analyzed by a particle size analyzer; typical size distribution of microcapsules prepared with a stirring speed at 400 rpm is shown in Figure 7. Then, the effect of stirring speed in the synthesizing process on the mean size ($D_{4,3}$) and size distribution width (SPAN) of the microcapsules prepared under different rates (300, 350, 400 and 500 rpm) was studied. As can be clearly seen from Figure 8, with the increase of stirring speed, the volume-based mean diameter ($D_{4,3}$) of the synthesized microcapsules decreased significantly from 352.2 to 218.5 μm. Simultaneously, the size distribution becomes narrow when the stirring rate increases, which can be reflected from the decrease of SPAN value from 1.667 to 0.831. These results indicate that the mean diameter and the size distribution of microcapsules can be controlled by varying the stirring speed during the polymerization process. Similar results have been reported [21].

### 3.4. Micromechanical Properties of Single Microcapsule

For mechanical trigger microcapsules, the understanding of the micromechanical properties of single microcapsule is a fundamental and crucial aspect. In previous reports, parallel plate compression apparatus [22] is commonly used to investigate the mechanical properties. Parameters about the shell such as elastic modulus have been calculated using the data obtained by the compression force profiles with fractional deformation. While in the real case of cementitious material cracking, the tip of a crack is normally regarded as a point of concentrated stress. Therefore, the fracture force (or bursting force) of microcapsules acquired by parallel plate compression cannot be used to simulate and investigate the trigger behavior of microcapsules under the strike of the crack. To understand the mechanical properties and trigger behavior of microcapsules in a more realistic way, a Berkovich indenter and nanoindentation technology are used to characterize the mechanical properties of a single microcapsules in this study. The elastic modulus of the shell and the rupture force of microcapsules is calculated directly by the software of the nanoindentation system.

#### 3.4.1. Elastic Modulus

**Figure 9:**
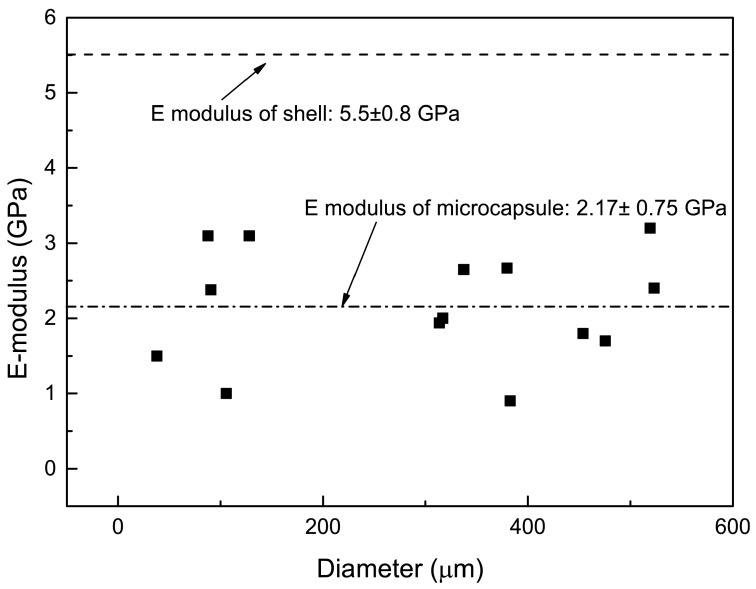
Elastic modulus of shell material and PF microcapsules, and its relationship with the diameter of microcapsules.

The elastic modulus of shell material and PF microcapsules was obtained by performing a nanoindentation test on polished shell material and a single microcapsule respectively. Figure 9 shows the mean E modulus of microcapsules and shell materials. No relationship was found between the E-modulus of microcapsules and its diameter. The mean E modulus of microcapsules is 2.2 ± 0.8 GPa which is significantly lower than that of pure shell materials with the value of 5.5 ± 0.8 GPa. The possible reason for this difference can be attributed to the deformation of microcapsules during the measurement and can also be partly attributed to the microcapsule being pushed into the glue. More information on structural effects when testing capsules with nanoindentation can be found in reference [23,24]. In this test, the Continuous Stiffness Method developed by Oliver and Pharr [20] was used. The basic idea of this method consists of superimposing a small displacement on the primary loading signal and, in real time, analyse the response of the system by using a frequency-specific amplifier. The E modulus is obtained by a continuous measure of contact stiffness (S) as a function of indentation depth (h). When the nanoindentation test is performed on the microcapsule, the indent force which is imposed on microcapsules will not only result in the Berkovich tip penetrating into the surface, but also makes the microcapsule have a slight deformation, which will finally increase the indentation depth and further decrease the value of the elastic modulus. The porosity of the shell will also result in differences in properties of the microcapsules, which is, for instance, reflected by a scatter in the E-modulus. To better understand the correlation between the E-moduli and the properties of microcapsules, such as diameter and shell thickness, a numerical modeling method will be used in a later stage.

#### 3.4.2. Rupture Force and Trigger Sensitivity Investigation

**Figure 10:**
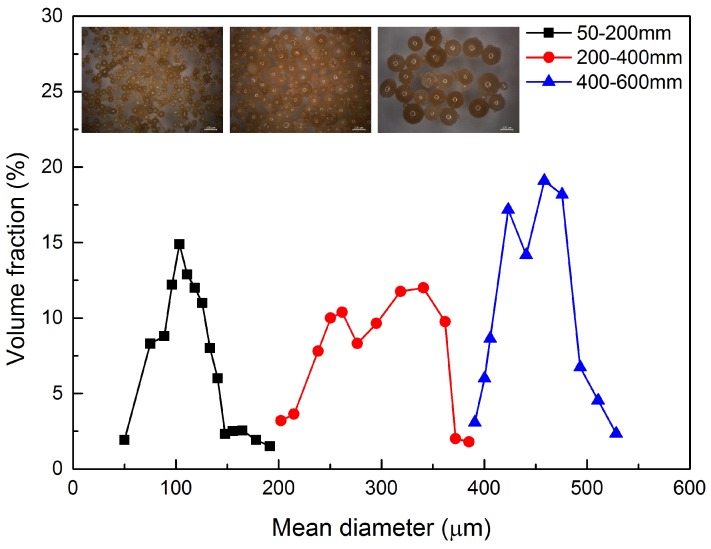
Size distribution of the PF microcapsules screened by sieves with various mesh sizes (30, 40, 70).

A microcapsule embedded in self-healing cementitious materials will fracture when a crack encounters that microcapsule. The trigger force which is imposed on the microcapsules can vary due to the nature and the width of the crack. To increase the healing efficiency and prolong the working life, it is ideal that the microcapsules have a different trigger sensitivity based on crack width. Consequently, microcapsules can be triggered accordingly to cracks with different crack opening. From previous research, it is known that the bursting force of microcapsules is related to their diameter [22]. In this respect, to achieve the multi-scale trigger function of cementitious material, the application of microcapsules with different diameters is essential. In this study, a sieve method was used to grade the synthesized microcapsules into several interval scales. Thanks to the excellent dispersibility and mechanical properties of the synthesized PF microcapsules, a gradation of microcapsules was successfully achieved. Figure 10 shows the size distribution of the PF/DCPD microcapsule after sieving made under the stirring rate of 400 rpm. As can be seen from the Figure, by passing the microcapsules through a series of sieves with mesh sizes of 30, 40, 70 (590, 420 and 210 μm in diameter), the particle size of the synthesized microcapsules ranging from 50 to 550 μm has been isolated into three intervals: small size (50–200 μm), medium size (200–400 μm) and large size (400–600 μm) respectively. Inserts gives the OM images of the sieved microcapsules; they are in accordance with those three size distribution regions shown below.

**Figure 11:**
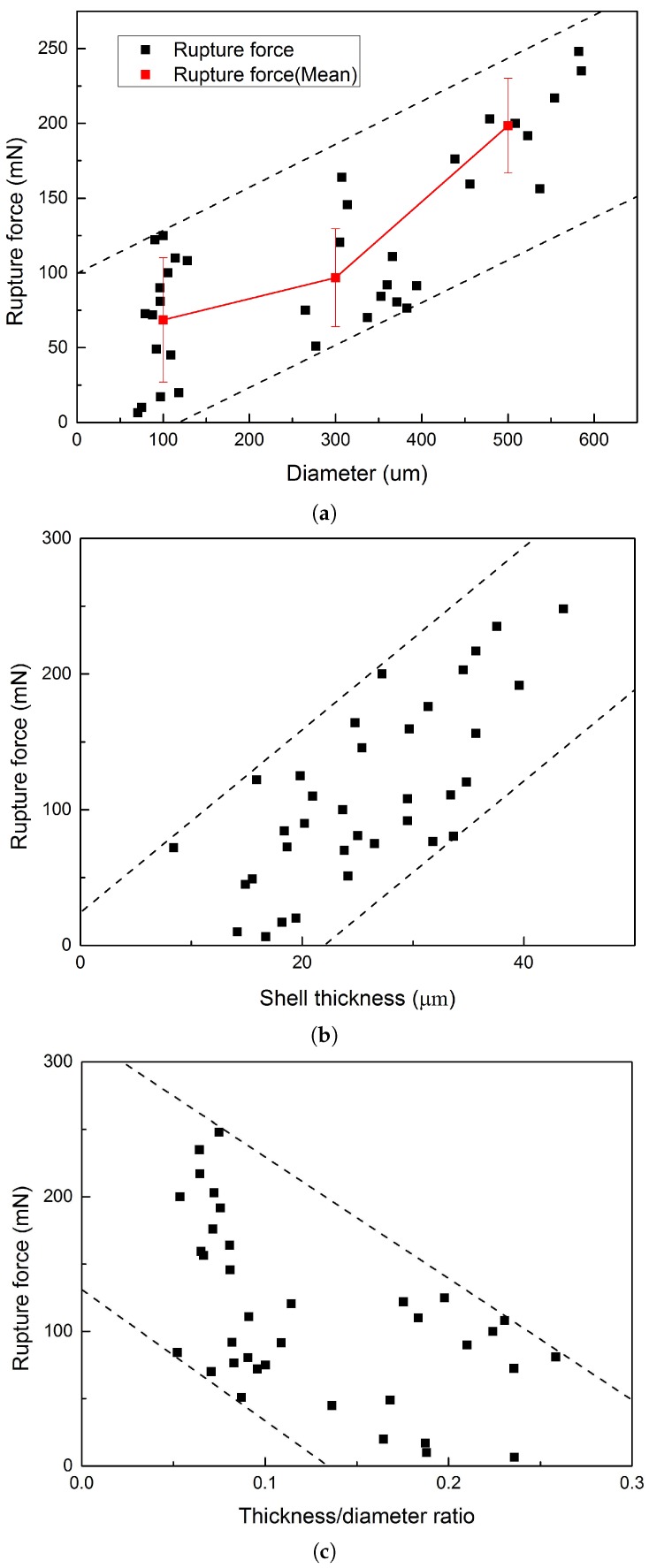
The relationship between the rupture force of PF microcapsules and their (**a**) diameter; (**b**) shell thickness; and (**c**) thickness/diameter ratio.

For further investigating the trigger sensitivity of microcapsules at different size gradations, the rupture force of single microcapsules was measured by the nanoindentation. Before the test, the diameter of a single microcapsule was measured under the build-in microscope. Figure 11a shows the relationship between the rupture force and the diameter of microcapsules. Specifically, the mean rupture force for small size (50–200 μm) microcapsules is 68.5 ± 41.6 mN. This number goes up to 96.8 ± 23.5 mN for medium size (200–400 μm) microcapsules. With the increase of diameter to the range of large size (400–600 μm) microcapsules, the rupture force of microcapsules increased continuously. The mean rupture force of large size microcapsules is 198.5 ± 31.6 mN. Despite the scatter, the rupture force shows an increasing trend with the diameter, a similar behavior can be found in a previous study for thin-shell PMMA microcapsules [22]. After the indentation test, the shell thickness of the ruptured capsules was measured under optical microscopy. Then, the relationship between the rupture force and the shell thickness of microcapsules was determined. Figure 11b shows the relationship between the rupture force and the shell thickness. As we can see from the figure, the rupture force which is required to break the capsules increases with the capsule shell thickness. In general, it can be concluded that both the increase of capsule size and shell thickness do have an influence on the trigger sensitivity of microcapsules. The bigger the size or the thicker the shell of the capsule is, the larger the force that will be needed to break it. These properties of the synthesized microcapsule can be used for the self-healing system, in which the capsules can react according to the strength of material or the width of the crack.

Finally, the rupture force was tried to correlate with the shell thickness/diameter ratio (Figure 11c). Unlike previous research for MF microcapsules [21], a heavily scattered decreasing trend was observed between the rupture force and shell thickness/diameter ratio. The possible reason for this decrease comes from the different growth rates of shell thickness and diameter. With the increase of capsule diameter, bigger capsules will normally have a relatively lower shell/diameter ratio. It is known that bigger microcapsules need a larger force to be ruptured. Thus, for bigger microcapsules with a low shell/diameter ratio, it is reasonable to have a higher rupture force and vice versa.

### 3.5. Influence of Microcapsules’ Incorporation on Mechanical Properties of Hardened Cement Paste

**Figure 12:**
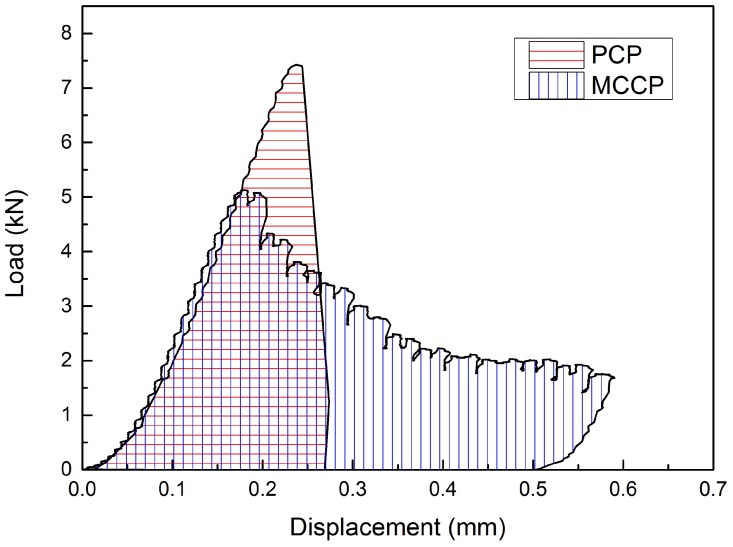
Load–displacement curves of a cement paste sample with and without microcapsules embedded.

In order to investigate the influence of microcapsules’ incorporation on the mechanical properties of cement paste, a compression test was performed on microcapsules-incorporated cement paste (MCCP) samples with a dimension of 10 × 10 × 10 mm^3^. The result of the above tests was then compared with pure cement paste (PCP). Figure 12 shows the typical load–displacement curves obtained from a cement paste sample with and without embedded microcapsules. For the pure cement paste, a familiar brittle fracture with low plastic deformation was observed. However, the capsule-embedded samples undergo an observable plastic-like deformation, so a more ductile behaviour was obtained. By incorporation of 4% microcapsules into cement paste, the strength decreased 32% from 7.4 kN to around 5 kN. The strength decrease is similar to that reported by Wang et al. [25] when they used the same volume of microencapsulated bacterial spores. On the other hand, Kanellopoulos et al. [26] observed no significant loss of compressive strength for less than 12% of microcapsules. This means, in short, that the mechanical properties of the composite are highly dependent on the capsule type used and should be tested for each new capsule type.

### 3.6. Crack-Zone Investigation by X-ray Computed Tomography

**Figure 13:**
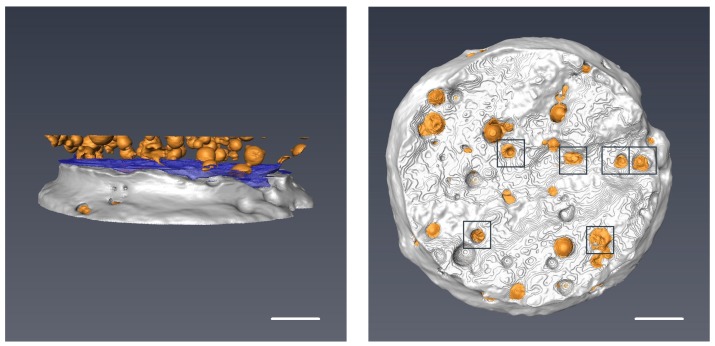
3D reconstructed images of the segmented raw data showing the crack (blue), microcapsules (yellow) and cement (grey); (**a**) a selected section of fractured cement paste in the vicinity of the crack; (**b**) a top-down view of crack surface. Ruptured microcapsules are indicated by a black square box. The scale bar is 1 mm for both images.

X-ray computed tomography (XCT) is a versatile inspection technique which has been widely applied in numerous fields of research [27,28,29,30]. Recently, the application of this technique has also been extended to the field of civil engineering [31,32,33,34]. As a non-destructive imaging technique, XCT provides an approach to obtain the internal information of a material [35]. In this study, XCT imaging technique combined with data reconstruction and image segmentation software was used to visualize the trigger behavior of the microcapsules-incorporated hardened cement paste specimens which are tested in tension. To investigate the rupture behavior of microcapsules along the crack zone, a small region of the cylinder adjacent to the crack was selected as the ROI (Region of interest). Then, different materials of the reconstructed segments were color labeled. Figure 13a shows the 3D representation of the ROI, where the microcapsules, the crack and the cement were defined as yellow, blue and grey color, respectively. The spatial dispersion of microcapsules along the crack demonstrates that the synthesized PF microcapsules can not only be well dispersed but also kept stable in a cement paste specimen. For further visualizing the crack surface of the ruptured sample, the upper half of the crack zone was isolated manually from the ROI. Figure 13b shows the top-down view of the segmented crack surface. As can be seen from the figure, when the crack goes through, a portion of the microcapsules was mechanically triggered by the crack. Meanwhile, there are still a large amount of microcapsules which cannot be triggered, leaving voids and untriggered microcapsules at the crack. It is believed that the voids come from the microcapsules which do not break but are pulled out of the matrix, where the crack goes around rather than through the microcapsules.

By inspecting the crack surface of cement paste samples containing (1) large size (400–600 μm); (2) medium size (200–400 μm); and (3) small size (50–200 μm) microcapsules, the statistic result of the triggered, untriggered microcapsules (MC) and the voids were obtained and shown in Table 1. It was found from the table that—for the large size microcapsules (400–600 μm) embedded cement paste sample—only 8.4% of those microcapsules were triggered on the path of crack. With the decrease of diameter from large size to medium size (200–400 μm), the trigger ratio of microcapsules increased sharply from 8.4% to 20.7%. Then, this number goes up to 34.7% when the size of embedded microcapsules decreases from the 200–400 μm to 50–200 μm. These results, together with the relationship between rupture force and diameter obtained above, demonstrate that the size distribution of microcapsules will influence the trigger efficiency of a capsule-based self-healing system. Smaller sized synthesized PF microcapsules (50–200 μm) tend to be more easily ruptured by the crack in cement paste.

## 4. Conclusions

In this study, PF microcapsules containing a healing agent were synthesized by the in situ polymerization method. Micromechanical properties of the synthesized microcapsules, including elastic modulus and rupture force, were measured using nanoindentation. Size gradation of synthesized microcapsules was achieved through a series of sieving processes. The mean shell thickness of microcapsules was found to have an increasing trend with the diameter, which was accurately measured under environmental scanning electron microscopy (ESEM). The mechanical result shows that with the increase of the mean size of microcapsules and the decrease of shell thickness, the mechanical force which is required to trigger the microcapsules increased correspondingly. In addition, the rupture behavior of the PF microcapsules was directly observed and analyzed using X-ray computed tomography (XCT) based on a microcapsule-embedded cement paste sample. Finally, the crack surface of the sample was inspected on the 3D reconstructed images and the rupture ratio of the embedded microcapsules was calculated. The result demonstrated that smaller sized synthesized PF microcapsules tend to be more mechanically triggered by the crack.

This work contributes to the search for an effective procedure to evaluate the micromechanical properties of mechanical trigger, self-healing, microcapsules and sheds light on the implementation of multi-sensitive self-healing materials. Further work is needed to improve the trigger efficiency of microcapsules. Meanwhile, with the data obtained above, a numerical simulation of the failure mechanism will be adopted to give more insight into the relation between rupture force, capsule diameter and shell thickness.

## Figures and Tables

**Table 1:** Trigger ratio microcapsules along the crack of cement paste with a diameter (1) 400–600; (2) 200–400; and (3) 50–200 μm.

Sample Number	Ruptured MC	Unruptured MC + Voids
1	8.6%	91.4%
2	20.7%	79.3%
3	34.7%	65.3%
